# Supplementary material for: Harnessing the LdCsm RNA Detection Platform for Efficient microRNA Detection
Source: Int J Mol Sci. 2023 Feb 2;24(3):2857. doi: 10.3390/ijms24032857 (PMC9918065; doi:10.3390/ijms24032857)
Supplement: Supplementary file 1 [file ijms-24-02857-s001.zip › ijms-2157199-supplementary.pdf]

This file includes:

- Figures S1
- Tables S1 to S3

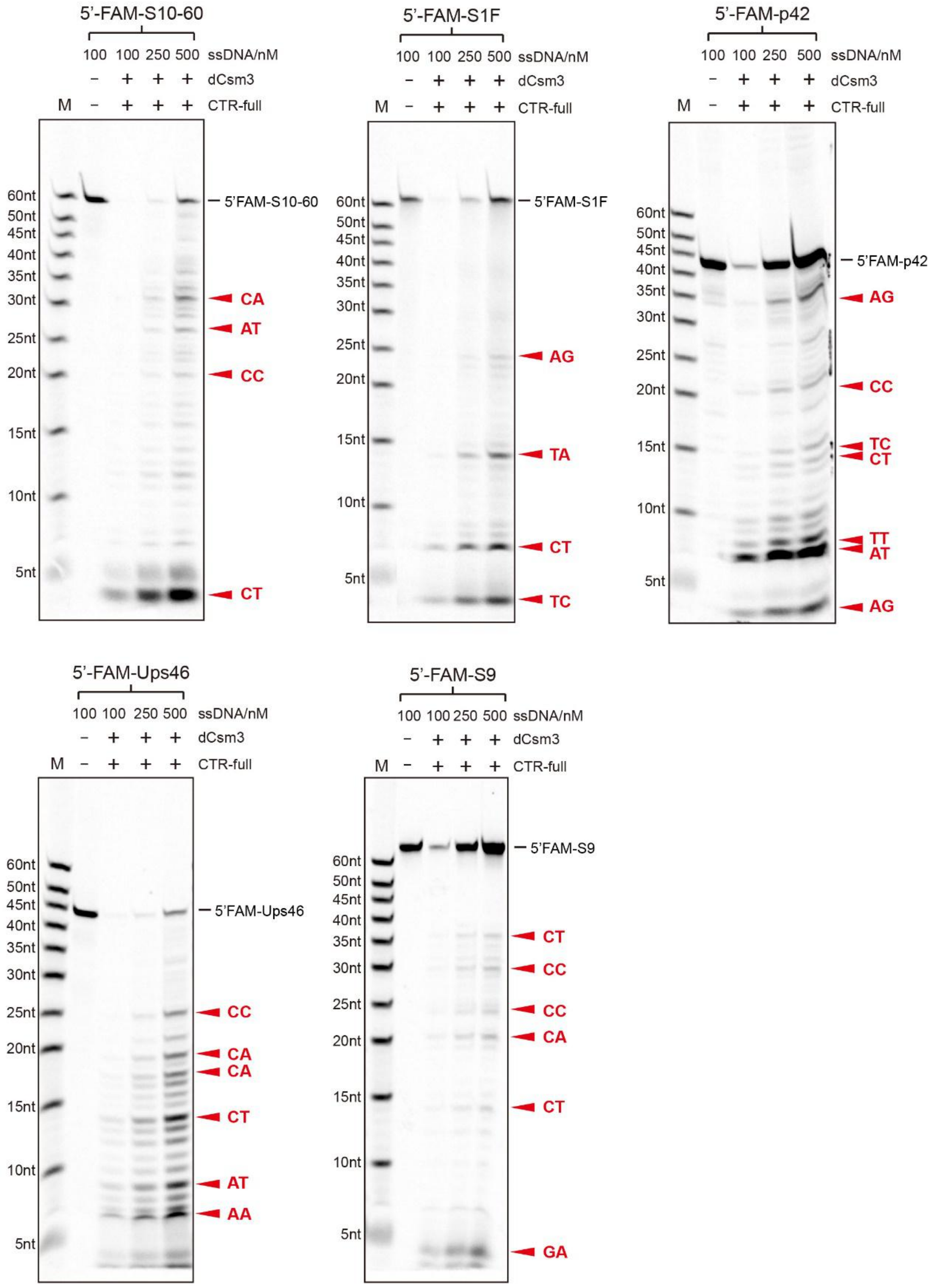

Figure S1. Investigation of base preference of LdCsm-dCsm3 DNase. Related to Figure 3

Denaturing PAGE of cleaved products of five different ssDNA substrates treated by target RNA-activated LdCsm-dCsm3. Locations of the main cleaved products of each ssDNA substrate are labeled by red triangle. Names of the main dinucleotide cleavage sites are shown on the right of the cleaved products.

Table S1 ssDNA primers and substrates used in this study.

ssDNA	Sequence(5'-3')	Description
**Primers**		
pUCE-repeat-F	GCTAGAAGGAGACGAGAACAAGCTTGCGGCCGCACTC	pUCE-S-155-1/2 construction
pUCE-repeat-R	GTTCTCGTCTCCTTCTAGCGGAGATAAGTTGTTAGCTTAATGCTAA	pUCE-S-155-1/2 construction
miR155-1-F	GCTAACAACTTATCTCCGCTAGAAGGAGACGAGAACCCTATCACGA	pUCE-S-155-1 construction
miR155-1-R	GATAAGTTGTTAGCTTAATGCTAATCGTGATAGGTTCTCGTCTCCTT	pUCE-S-155-1 construction
miR155-1-A−3U-F	GCTAACAACTTATCTCCGCTAGAAGGAGACGAGTACCCTATCACGA	pUCE-S-155-1-A−3U construction
miR155-1-A−3U-R	AGATAAGTTGTTAGCTTAATGCTAATCGTGATAGGGTACTCGTCTC	pUCE-S-155-1-A−3U construction
miR155-2-F	GCTAACAACTTATCTCCGCTAGAAGGAGACGAGAACCTATCACGAT	pUCE-S-155-2 construction
miR155-2-R	GATAAGTTGTTAGCTTAATGCTAATCGTGATAGGTTCTCGTCTCCTT	pUCE-S-155-2 construction
miR155-2-A−5U-F	GCTAACAACTTATCTCCGCTAGAAGGAGACGTGAACCTATCACGAT	pUCE-S-155-2-A−5U construction
miR155-2-A−5U-R	GATAAGTTGTTAGCTTAATGCTAATCGTGATAGGTTCACGTCTCCTT	pUCE-S-155-2-A−5U construction
pUCE-seq-F	GTAAACTTGGTCTGACAGGCTA	pUCE plasmids sequencing
pUCE-seq-R	GCTGCAGATCCGGATATAGTTCCTCCT	pUCE plasmids sequencing
**ssDNA substrates**		
FAM-S10-60	ACTATAGGGAGAATAGAATGCCCCCATTATACAATATCTACGTTTTAGA TGACCCCCCCC	5' FAM labeled ssDNA substrate
FAM-S1F	TTCGAGCTCGGTACCCGGTTGATAGGGACAAGACGAATAGGGGATCCTC TAGAGTCGAC	5' FAM labeled ssDNA substrate
FAM-S1R	GTCGACTCTAGAGGATCCCCTATTCGTCTTGTCCCTATCAACCGGGTACC GAGCTCGAA	5' FAM labeled ssDNA substrate
FAM-p42	CAGTGAATTCGAGCTCGGTACCCGGGGATCCTCTAGAGTCGA	5' FAM labeled ssDNA substrate
FAM-Ups46	TCAGAAAAAATATACTCACACAATACCGAAATTACTTAAAACTTCC	5' FAM labeled ssDNA substrate
FAM-p35	GTGACAGCATCTCATACTCCAGAGGCTAAGGTGAA	5' FAM labeled ssDNA substrate
FAM-S9	ACAGATGGGCGTTCTGGAGGCAGCCGAGTCCGTATCTCTTCCTGAAAGG GAGGGAGAAGGTTGT	5' FAM labeled ssDNA substrate
poly-T reporter	TTTTTTTTTTTTTTTT	5' FAM labeled and 3' BHQ1 labeled fluorescent quenched ssDNA reporter
CT reporter	CTCTCCTCCTTCTTC	5' FAM labeled and 3' BHQ1 labeled fluorescent quenched ssDNA reporter

Table S2 Statistic of deduced main dinucleotide cleavage sites in seven ssDNA substrates. Related to Figure 3.

ssDNA substrate \ Main cleavage sites	CT	CC	CA	AT	AG	AA	TC	TA	TT	GA	All
S10-60	1	1	1	1							4
S1F	1				1		1	1			4
S1R	3	3									6
p42	1	1		1	2		1		1		7
Ups46	1	1	2	1		1					6
p35	2	1	3								6
S9	2	2	1							1	6
Total in 7 ssDNA substrates	11	9	7	3	3	1	2	1	1	1	39

Preferred cleavage sites of the LdCsm-dCsm3 DNase are highlighted in red.

Table S3 RNA substrates used in this study.

RNA	Sequence(5'-3')	Description
S10	AUAGAAUGCCCCCAUUAUACAAUAUCUACGUUUUAGAUGAAAAAAA	Unspecific RNA
Cas13a crRNA	GGGGAUUUAGACUACCCCAAAAACGAAGGGGACUAAAACUUCAAAGC UUAGAUACCCUGGAGG	crRNA of LwaCas13a with S1 spacer
RNA reporter	UUUUUU	5' FAM labeled and 3' BHQ1 labeled fluorescent quenched RNA reporter
miR-155	UUAAUGCUAAUCGUGAUAGGGGUU	Breast cancer-related microRNA
miR-124	CGUGUUCACAGCGGACCUUGAU	Human microRNA
miR-149	UCUGGCUCCGUGUCUUCACUCCC	Human microRNA
miR-340	UUAUAAAGCAAUGAGACUGAUU	Human microRNA
miR-375	GCGACGAGCCCCUCGCACAAACC	Human microRNA
**Target RNA of S1 spacer**	**Sequence(3'-5')**	
PTR	AAGUUUCGAAUCUAUGGGACCUCCCUUUGGUCUGAAUUGU	Target RNA with no 3′anti-tag
PTR+1	AAAGUUUCGAAUCUAUGGGACCUCCCUUUGGUCUGAAUUGU	Target RNA with 1 A in 3′anti-tag
PTR+2	AAAAGUUUCGAAUCUAUGGGACCUCCCUUUGGUCUGAAUUGU	Target RNA with 2 A in 3′anti-tag
PTR+3	AAAAAGUUUCGAAUCUAUGGGACCUCCCUUUGGUCUGAAUUGU	Target RNA with 3 A in 3′anti-tag
PTR+4	AAAAAAGUUUCGAAUCUAUGGGACCUCCCUUUGGUCUGAAUUGU	Target RNA with 4 A in 3′anti-tag
PTR+5	AAAAAAAGUUUCGAAUCUAUGGGACCUCCCUUUGGUCUGAAUUGU	Target RNA with 5 A in 3′anti-tag
PTR+6 (CTR-full)	AAAAAAAAGUUUCGAAUCUAUGGGACCUCCCUUUGGUCUGAAUUGU	Target RNA with 6 A in 3′anti-tag
CTR34	AAAAAAAAGUUUCGAAUCUAUGGGACCUCCCUUUGGUCUGAA	CTR with 34nt in protospacer
CTR29	AAAAAAAAGUUUCGAAUCUAUGGGACCUCCCUUUG	CTR with 29nt in protospacer
CTR23	AAAAAAAAGUUUCGAAUCUAUGGGACCUC	CTR with 23nt in protospacer
CTR22	AAAAAAAAGUUUCGAAUCUAUGGGACCU	CTR with 22nt in protospacer
CTR21	AAAAAAAAGUUUCGAAUCUAUGGGACC	CTR with 21nt in protospacer
CTR20	AAAAAAAAGUUUCGAAUCUAUGGGAC	CTR with 20nt in protospacer
CTR19	AAAAAAAAGUUUCGAAUCUAUGGGA	CTR with 19nt in protospacer
CTR18	AAAAAAAAGUUUCGAAUCUAUGGG	CTR with 18nt in protospacer
CTR17	AAAAAAAAGUUUCGAAUCUAUGG	CTR with 17nt in protospacer
CTR16	AAAAAAAAGUUUCGAAUCUAUG	CTR with 16nt in protospacer
CTR15	AAAAAAAAGUUUCGAAUCUAU	CTR with 15nt in protospacer
CTR14	AAAAAAAAGUUUCGAAUCUA	CTR with 14nt in protospacer

CTR13	AAAAAAAAGUUUCGAAUCU	CTR with 13nt in protospacer
CTR12	AAAAAAAAGUUUCGAAUC	CTR with 12nt in protospacer
CTR11	AAAAAAAAGUUUCGAAU	CTR with 11nt in protospacer

3'-antitags of CTR are colored in red.
